# Dental Sealants: Knowledge, Value, Opinion, and Practice among Dental Professionals of Bathinda City, India

**DOI:** 10.1155/2014/469738

**Published:** 2014-04-10

**Authors:** Kailash Asawa, Vivek V. Gupta, Mridula Tak, Ramesh Nagarajappa, Pulkit Chaturvedi, Salil Bapat, Prashant Mishra, Santanu Sen Roy

**Affiliations:** ^1^Department of Public Health Dentistry, Pacific Dental College and Hospital, Debari, Udaipur, Rajasthan, India; ^2^Department of Public Health Dentistry, Rama Dental College and Hospital, Kanpur, Uttar Pradesh, India; ^3^Department of Public Health Dentistry, Sri Aurobindo Institute of Dental Sciences, Indore, Madhya Pradesh, India; ^4^Department of Public Health Dentistry, Guru Nanak Dental College & Hospital, Kolkata, West Bengal, India

## Abstract

*Objective*. The purpose of the study was to assess the knowledge, value, opinion, and practice regarding use of dental sealants among private dental practitioners in Bathinda City, Punjab, India. *Materials and Methods*. A cross-sectional survey was conducted among all private dental practitioners in Bathinda City, Punjab. A self-administered structured questionnaire consisting of 28 items was used to assess their knowledge, value, opinion, and practice regarding dental sealants. One-way analysis of variance, independent sample *t*-test, and multivariate regression analysis were utilized for statistical analysis. Confidence level and level of significance were set at 95% and 5%, respectively. *Results*. The mean scores for knowledge, value, opinion, and practice were 41.8 ± 3.7, 18.7 ± 2.8, 18.1 ± 1.4, and 12.9 ± 2.3, respectively. Analysis revealed that qualification was statistically significant among all dependent variables (*P* ≤ 0.05); work experience was significantly associated with both knowledge and opinion means scores (*P* ≤ 0.05). *Conclusion*. The results suggest that dental practitioners had sufficient knowledge about dental sealants. They also acknowledge the importance of use of dental sealants. Practice of dental sealants in clinics was found adequate but they were not following the specific guidelines and standardized procedures.

## 1. Introduction

Dental caries is an important public health problem and it is the most prevalent oral disease among children. This disease not only causes damage to the tooth but is also responsible for several morbid conditions of the oral cavity and other systems of the body [1]. The global distribution of dental caries has shown distinctive variations [2]. The scenario in India also shows similarities with other developing countries. A very extensive and comprehensive National Health Survey conducted in 2004 throughout India has shown dental caries prevalence of 51.9% in 5-year-old children, 53.8% in 12-year-old children, and 63.1% in 15-year-old teenagers [3].

A survey conducted in 7 districts (Arunachal Pradesh, Delhi, Maharashtra, Puducherry, Rajasthan, Orissa, and Uttar Pradesh), covering 3200 subjects from each site, showed a very high prevalence of dental caries in Puducherry (71.5%), while Orissa was found to have the least prevalence (23%) among the 12-year-old age group [4]. Apart from these, the anecdotal prevalence has been obtained at various locations in Punjab. In the year 2013, the prevalence of dental caries in Ludhiana was found to be as high as 81.4% in the 12-year-old age group and 86.2% among 15-year-old age group [5]. In a similar study among 5- and 12-year-old schoolchildren of Chandigarh the prevalence of dental caries was found to be 48.3% and 30.5% in the year 2012 [6].

Pits and fissures are the most caries-vulnerable sites of teeth; the percentage of total caries attributable to occlusal surfaces compared with smooth surfaces has increased [7]. It was generally accepted that nearly all molar occlusal surfaces would eventually become carious [8–10]. As reviewed by Eklund and Ismail, during the 1950s, 1960s, and 1970s, 70% of all molar occlusal surfaces became carious within 10 years of emergence into the oral cavity [11].

Buonocore's classic study of 1955 marked the start of a major revolution in the clinical practice of dentistry [12]. The first clinical benefit from Buonocore's work was the introduction of the first dental pit and fissure sealant, Nuva-Seal (L.D. Caulk) in February 1971 [13]. The term pit and fissure sealant is used to describe a material that is introduced into the occlusal pits and fissures of caries-susceptible teeth, thus forming a micromechanically bonded, protective layer cutting access of caries-producing bacteria from their source of nutrients [14].

Strong evidence shows that sealants are effective in preventing caries [15, 16]. Over the last few years more than 11 guidelines and systematic reviews have recommended pit and fissure sealant use for at-risk populations [17–19]. However, more than 30 years after the introduction of pit and fissure sealant to the dental market place, the profession has not embraced the procedure to the extent that available scientific data would expect [13]. Studies from USA [20], Greece [21], Sweden [22], and Scotland [23] all indicate that sealants are underutilized. Hence, the objective of the present cross-sectional study was to assess the knowledge, value, opinion, and practice (KOVP) regarding dental sealants among dental professionals of Bathinda City, Punjab, India.

## 2. Materials and Methods 

### 2.1. Study Design and Population

A descriptive cross-sectional study was conducted among dental professionals of Bathinda City, Punjab, India, in the month of July 2013. Study population consisted of all the private dental practitioners of Bathinda City.

### 2.2. Ethical Approval

The study protocol was reviewed by the Ethical Committee of Pacific Dental College and Hospital and was granted ethical clearance.

### 2.3. Pretesting of Questionnaire

A self-administered structured questionnaire was developed and tested among a convenience sample of 10 dentists, who were interviewed to gain feedback on the overall acceptability of the questionnaire in terms of length and language clarity. Based on their feedback, the questionnaire did not require any corrections. Cronbach's coefficient was found to be 0.80, which showed an internal reliability of the questionnaire. Mean content validity ratio (CVR) was calculated as 0.87 based on the opinions expressed by a panel of five academicians. Face validity was also assessed and it was observed that 92% of the participants found the questionnaire to be easy.

### 2.4. Questionnaire

The questionnaire, designed to obtain dentists' knowledge, opinion, value, and practice towards dental sealants, consisted of five sections. Section I solicited general demographic details and information on professional background. The dentists involved in active patient care were asked about years of experience in a dental clinic. Section II integrated 11 questions to collect information about* knowledge* regarding dental sealants. Section III comprised of 5 questions which aimed to assess the* value* regarding dental sealants. Section IV contained 5 questions regarding* opinion* about dental sealants. Section V consisted of 7 questions regarding dental sealants* practice*. The participant's responses for Sections II, III, and IV were ranked according to how much they agreed with each statement that was based on the 5 point Likert scale with alternatives: strongly disagree, disagree, neutral, agree, and strongly agree. For Section V the participant's responses were recorded as never, sometimes, and always.

### 2.5. Methodology

The investigator collected the list of private practicing dentists from local sources (local IDA branch and telephone directory). Among a total of 166 dental practitioners, a pilot study was conducted on 10 dental practitioners. These were later excluded from the main study and the final sample size was 156 practitioners. On the predecided days, the investigator visited the private clinics, according to area of distribution, to get the questionnaire filled. Questionnaires were distributed among all dentists (*n* = 156) who were requested to fill in the written informed consent form and were asked to rate each item of the questionnaire choosing the most appropriate response. The investigator revisited the clinics after 3 days to collect the filled questionnaires. Hundred percent response rate was achieved by 2-3 followups.

### 2.6. Statistical Analysis

Completed questionnaires were coded and spreadsheets were created for data entry. The data was analyzed using SPSS 15 (SPSS Inc., Chicago, IL, USA) Windows software program.

Responses to all items of the Sections II, III, and IV were coded from 1–5 (strongly disagree to strongly agree). Responses in Section V were coded from 1–3 (never, sometimes, and always). Several items like question 9 in Section II, question 1, 2, and 3 in Section III, and questions 1, 2, and 5 in Section IV were recoded to ensure that a high score indicated a positive knowledge, value, opinion, and practice and a low score indicated a negative knowledge, value, opinion, and practice. Mean knowledge, opinion, value, and practice scores and standard deviation were calculated. For frequency distribution, strongly disagree and disagree responses were combined into one category (disagree) and similarly agree and strongly agree responses (agree). Descriptive statistics were used to summarize the demographic information and the survey data was analyzed using the student's *t*-test and one-way ANOVA with post hoc Bonferroni test. Multivariate logistic regression analysis models were used to check relation between independent (age, sex, qualification, and work experience) and dependent variables (knowledge, value, opinion, and practice). Confidence level and level of significance were fixed at 95% and 5%, respectively.

## 3. Results 

In Table 1 a total of 156 dentists with the mean age (in years) of  32.9 ± 5.3 participated in the survey. Demographic data showed that the majority of the respondents were graduates (65.4%), males (59%), and in the age group of 25–35 years (71.2%). 53.2% of the dental health professional had <5 years of work experience.

In Table 2 the mean knowledge, value, opinion, and practice scores of the study population were evident as  41.8 ± 3.7, 18.7 ± 2.8, 18.1 ± 1.4, and  12.9 ± 2.3, respectively. Postgraduates had significantly greater knowledge and practice than graduates. Mean scores for postgraduates and graduates were 44.5 ± 2.9, 39.7 ± 2.9 (*P* = 0.000) and 13.7 ± 0.4, 12.5 ± 2.7 (*P* = 0.01), respectively. When post hoc Bonferroni test was applied, mean knowledge score among those who had less than 5 years of experience (42.3 ± 4.2) was found to be significantly greater than among those who had more than 10 years of experience (40.7 ± 3.5) (*P* = 0.001).

Table 3 depicted that 84.6% of the dentists showed familiarity with the sealant placement technique and 84.7% believed that sealants should be reviewed after placement. 62.8% of the dentists thought that materials used for the placement of sealants are very expensive and their effect is very short lived. 80.2% of the dentists found difficulty in justifying the cost of sealants to the parents and 78.8% of the dental professionals had an opinion that it is necessary to promote the use of sealants among dentists and dental educators.

In Table 4 multivariate logistic regression analysis revealed that knowledge had a significant association with qualification and years of work experience [OR = 2.5 (0.4–5.0), OR = 0.9 (0.6–5.1)] and practice showed a significant statistical association with qualification [OR = 0.8 (0.2–1.9)], respectively.

## 4. Discussion 

The present study was conducted among 156 dental practitioners of Bathinda City, Punjab, to assess their dental sealants knowledge, value, opinion, and practice. To the best of our knowledge, this is the first study to examine the knowledge, value, opinion, and practice of dental sealants among Indian dental practitioners.

In the present study, knowledge, value, and opinion on dental sealants was found to be associated with years of work experience and qualification. Multivariate analysis revealed that knowledge showed significant association with qualification and years of work experience, while practice showed significant association with qualification only. Dental professionals who had more than 10 years of work experience had less knowledge than those who had 5–10 or less than 5 years of work experience and this is in accordance with a study conducted by San Martin et al. [24] among Spanish dentists. This might be due to lack of frequent practice of dental sealants in their clinical practice. The study also revealed that dentists with postgraduate qualifications showed a significantly greater mean knowledge and practice score than those with undergraduate qualifications. This might be due to their more familiarity and use of dental sealants when they were perusing their postgraduation degree in pediatric dentistry, restorative dentistry, and preventive dentistry [21].

In the present study, 52% of dentists responded that it was difficult to justify the cost of sealants to parents. This finding might be correlated to the study conducted by Albert et al. [25] who stated that pit and fissure sealant treatment plans offered by American dentists are rejected by 55.7% of the parents most frequently because of the cost factor. In contrast San Martin et al. [24] reported lesser proportion of Spanish dentists (34.6%) portraying difficulty in justifying the cost of sealants to the parents. Moreover in our study, around 62.8% of dental professionals agreed that materials used for placement of sealants were very expensive which is in contrast to the findings of San Martin et al. [24] among Spanish dentists (13.1%). This might be due to the fact that the National Health System at Spain provides care for all children, independent of parent income [24].

In contrast to 31.6% of Spanish dentists [24], 59% of the dental professionals in the present study depicted difficulty in explaining to patients what dental sealants are. This might be due to higher literacy rate and awareness among Spanish population regarding dental sealants.

In the present study, 35.9% of the study subjects agreed about the adverse effects of dental sealant and 35.9% showed a neutral response which shows considerable doubt in the minds of many dentists about the safety of pit and fissure sealants. No reports of adverse health effects have been attributed to the leached components of dental sealants. It is therefore questionable whether these materials indeed are leached out of dental sealants in quantities that can pose a health hazard [26].

84.6% of dental practitioners in our study were found to be familiar with sealant placement technique and 83.9% believed that proper technique of sealant application is fundamental to the success of the treatment. 87.2% of them were following proper isolation and acid etching techniques but contrary to this around 84.6% of them responded that they were not following specific guidelines. This may be attributed to the belief of 69.8% dentists that the technique takes time to do correctly [27].

62.8% of practitioners in our study responded that they do not use sealants as a preventive method very often because its effect is short lived and this may be attributed to the knowledge of the practitioners that the sealants wear off easily. Contrary to this 84.7% of the dentists believed that if at all they place sealants it should be reviewed periodically. High retention rates of sealants were achieved when recall was incorporated [28].

In our study, 78.8% of respondents agreed about the effectiveness of resin sealants over glass ionomer sealants [29]. The resin-based sealant is superior not only in terms of retention, but also in caries prevention [30].

Out of 156 dental professionals, approximately 80% were avoiding dental sealants for the possibility of sealing over caries. In contrast, [31] noted that a limited number of cultivable organisms persist in some lesions which do not appear capable of continuing the destruction of tooth structure.

78.8% of participants in this study found it necessary to promote the use of dental sealants. Professional organizations should take a more active role in promoting sealant use among dentists [32].

In the present study, significantly greater proportion (85.3%) of the participants found that better caries prevention can be attained when sealants were used along with fluorides. Additional caries-preventive benefits were observed when pit and fissure sealants were applied along with fluoride therapy [33]. It has been recognized that the addition of fluoride to a sealant or perhaps to the enamel prior to sealant application could have the potential benefit of additional caries protection, without compromising the properties of the sealant [34].

In case of partial or complete loss of sealants, 85.9% of dentists were not reapplying regardless of sealant retention; caries experience was low under partially retained or missing sealants and completely retained sealants [35, 36].

The present study surveyed all the private dental practitioners of Bathinda City with a 100% response rate. Moreover, the self-administered questionnaire used in the study was previously calibrated and validated for the present study population.

The assessment of knowledge, value, opinion, and practice was based on dental practitioners self-report. Questionnaires were administered among all the dental practitioners in the city to provide a more comfortable environment for the participants in which to answer the questions. Moreover, participants were assured that their responses would be solely used for this research. Limitations of the present study are that we are not sure how truthfully and thoughtfully the respondents answered the questionnaire and level of subjectivity is not acknowledged in the present study.

## 5. Conclusion

The present study concluded that dental practitioners had sufficient knowledge about dental sealants. They also acknowledge the importance of use of dental sealants. Practice of dental sealants in clinics was found adequate but they were not following the specific guidelines and standardized procedures. To overcome this, professional and government bodies should create clear strategies for enhancing and improving dental practitioner's knowledge and make them confident on suggesting and using sealants. This study opens new vista for more detailed research among other dental practitioners in other parts of the country.

## Figures and Tables

**Table 1:** Demographic characteristics of dentists of study population.

Sample characteristics	Frequency (%)
Age (in years)	
25–34	101 (64.7)
35–45	43 (27.6)
>45	12 (7.7)
Sex	
Male	92 (59)
Female	64 (41)
Qualification	
MDS	54 (34.6)
BDS	102 (65.4)
Years of experience (in years)	
<5	83 (53.2)
5–10	36 (23.1)
>10	37 (23.7)

Total	156 (100)

**Table 2:** Association of mean knowledge, value, opinion, and practice score with independent variables.

Variables	Knowledge		Value		Opinion		Practice	
	Mean ± SD	*P* value	Mean ± SD	*P* value	Mean ± SD	*P* value	Mean ± SD	*P* value
Sex								
Male	41.6 ± 3.7	0.47	17.4 ± 2.2	0.88	18.3 ± 2.3	0.07	12.9 ± 2.2	0.76
Female	41.1 ± 3.6		18.3 ± 1.9		18.1 ± 1.9		12.9 ± 2.5	
Age (in years)								
25–34	40.9 ± 3.7	0.06	17.6 ± 2.1	0.96	18.1 ± 2.1	0.11	12.8 ± 2.5	0.42
35–44	42.3 ± 3.5		17.7 ± 2.0		18.7 ± 2.0		13.3 ± 1.9	
≥45	42.4 ± 3.8		17.7 ± 2.3		19.1 ± 2.9		13.3 ± 2.0	
Qualification								
MDS	44.5 ± 2.9	0.00*	18.2 ± 1.9	0.07	20.2 ± 1.8	0.08	13.7 ± 0.4	0.01*
BDS	39.7 ± 2.9		17.7 ± 2.0		19.3 ± 1.6		12.5 ± 2.7	
Work experience (in years)								
<5	42.3 ± 4.2^a^	0.04*	17.7 ± 2.0	0.82	18.9 ± 2.0	0.78	12.8 ± 2.5	0.37
5–10	42.1 ± 3.3		17.8 ± 2.2		18.4 ± 1.9		12.9 ± 2.5	
>10	40.7 ± 3.5^a^		17.5 ± 2.1		17.8 ± 2.6		13.4 ± 1.7	

Total	41.8 ± 3.7		18.7 ± 2.8		18.1 ± 1.4		12.9 ± 2.3	

Statistical tests applied: *t* test, one way ANOVA.

*indicates statistically significant difference at *P* ≤ 0.05.

Post hoc Bonferroni test: Groups with same letter (a) suprascripted showed statistically significant difference.

**Table 3:** Frequency of responses regarding knowledge, value, opinion, and practice.

	Disagree	Neutral	Agree
Knowledge			
I think that the effectiveness of fissure sealants is supported by strong scientific evidence	0	35 (22.4)	121 (77.6)
There is scientific evidence for the restorative use of dental sealants	2 (1.2)	41 (26.3)	113 (72.5)
I am familiar with the technique of placing dental sealants	20 (12.7)	4 (2.6)	132 (84.7)
I believe that fissure sealants should be reviewed after placement	6 (3.8)	18 (11.7)	132 (84.7)
I understand the instructions for placing sealants	7 (4.5)	16 (10.3)	133 (85.2)
I think that sealants should only be used on newly erupted teeth	31 (19.9)	44 (28.2)	81 (51.9)
I think that sealants wear out easily	29 (18.6)	27 (17.3)	100 (64.1)
I believe that you must perform a caries risk assessment to prevent overtreatment	0	41 (26.3)	115 (73.7)
Pit and fissure sealants have adverse effects	44 (28.2)	56 (35.9)	56 (35.9)
I believe the technique of applying a sealant is the most important aspect to the success of the treatment	1 (0.6)	24 (15.4)	131 (83.9)
I agree that resin sealants are more effective than glass ionomer sealant	0	33 (21.2)	123 (78.8)
Value			
I think this technique takes time to do correctly	4 (2.5)	43 (27.6)	109 (69.8)
The materials that are used for the placement of sealants are very expensive	46 (29.5)	12 (7.7)	98 (62.8)
I do not use sealants very often as a preventive method because its effect is short lived	35 (22.4)	23 (14.7)	98 (62.8)
Fissure sealants are used less than they should be	14 (9.0)	19 (12.2)	123 (78.8)
The dental staff at my clinic/work place communicates importance of using sealants to the patients	3 (1.9)	30 (19.2)	123 (78.8)
Opinion			
It is difficult to explain to patients what dental sealants are	21 (13.4)	41 (26.3)	94 (60.3)
It is difficult to justify the cost of sealants to parents	21 (13.4)	10 (6.4)	125 (80.2)
I think my patients understand the benefits of using sealants	1 (0.6)	29 (18.6)	126 (80.8)
It is necessary to promote the use of sealants amongst dentists and dental educators	0	33 (21.2)	123 (78.8)
I use dental sealant because it is easy to apply and patients find it comfortable	2 (1.2)	59 (37.9)	95 (60.9)

	Never	Sometimes	Always

Practice			
Do not use due to possibility of sealing over caries	24 (15.4)	12 (7.7)	120 (76.9)
Use as restorative material to restore incipient caries	6 (3.8)	18 (11.5)	132 (84.6)
Use along with fluorides	7 (4.5)	16 (10.3)	133 (85.3)
Do not reapply in case of partial or complete loss	0	22 (14.1)	134 (85.9)
Proper isolation	0	20 (12.8)	136 (87.2)
Proper acid etching	0	20 (12.8)	136 (87.2)
Not following specific guidelines	5 (3.2)	19 (12.18)	132 (84.6)

**Table 4:** Multiple logistic regression odd ratios (95% CI) for knowledge, value, opinion, and practice score as dependent variable among dentists.

Variables	Age (<*45*/>45 years)	Sex (*male*/female)	Qualification (*BDS*/MDS)	Work experience (<*10*/>10 years)
Knowledge	1.8 (0.5–7.1)	0.7 (0.1–3.7)	2.5 (0.4–5.0)*	0.9 (0.6–5.1)*
Value	1.3 (0.6–2.9)	1.0 (0.4–2.7)	3.7 (1.8–7.6)	1.1 (0.5–3.3)
Opinion	1.6 (0.3–7.9)	0.6 (0.1–4.8)	0.2 (0.0–1.5)	2.6 (0.3–4.9)
Practice	1.2 (0.4–2.2)	0.2 (0.1–1.9)	0.8 (0.2–1.9)*	4.4 (0.7–6.2)

Italicized category is taken as reference group.
